# Intravital imaging reveals p53-dependent cancer cell death induced by phototherapy via calcium signaling

**DOI:** 10.18632/oncotarget.2935

**Published:** 2014-12-02

**Authors:** Carlotta Giorgi, Massimo Bonora, Sonia Missiroli, Federica Poletti, Fabian Galindo Ramirez, Giampaolo Morciano, Claudia Morganti, Pier Paolo Pandolfi, Fabio Mammano, Paolo Pinton

**Affiliations:** ^1^ Department of Morphology, Surgery and Experimental Medicine, Section of Pathology, Oncology and Experimental Biology and LTTA center, University of Ferrara, Ferrara, Italy; ^2^ Department of Physics and Astronomy, University of Padua, and Venetian Institute of Molecular Medicine, Padua, Italy; ^3^ Instituto de fisiologia, Benemerita Universidad Autónoma de Puebla (BUAP), Puebla, Mexico; ^4^ Cancer Genetics Program, Beth Israel Deaconess Cancer Center, Departments of Medicine and Pathology, Beth Israel Deaconess Medical Center, Harvard Medical School, Boston, MA, USA

**Keywords:** Calcium (Ca^2+^), cell death, apoptosis, TRP53 (p53), mitochondria

## Abstract

One challenge in biology is signal transduction monitoring in a physiological context. Intravital imaging techniques are revolutionizing our understanding of tumor and host cell behaviors in the tumor environment. However, these deep tissue imaging techniques have not yet been adopted to investigate the second messenger calcium (Ca^2+^). In the present study, we established conditions that allow the *in vivo* detection of Ca^2+^ signaling in three-dimensional tumor masses in mouse models. By combining intravital imaging and a skinfold chamber technique, we determined the ability of photodynamic cancer therapy to induce an increase in intracellular Ca^2+^ concentrations and, consequently, an increase in cell death in a p53-dependent pathway.

## INTRODUCTION

The development of malignant tumors results from the deregulated proliferation of cells or from the inability of cells to undergo apoptotic death [1]. Consequently, apoptosis induction in tumor cells is one of the aims of anti-cancer therapy. Studies during the past decade have highlighted the importance of calcium (Ca^2+^) signaling in the regulation of key aspects of cell death. In particular, the modulation of Ca^2+^ signaling can change cell sensitivity to apoptotic signals, such as chemotherapeutic agents [2].

Several studies have indicated that the Ca^2+^ content of the endoplasmic reticulum (ER) determines cell sensitivity to apoptotic stress and that perturbation of ER Ca^2+^ homeostasis appears to be a key component in the development of several pathologies. Therefore, changes in the Ca^2+^ flux are regulated by Ca^2+^ release from the ER store toward the cytosol, and mitochondria can promote the survival of cancer cells, reducing sensitivity to apoptotic signals [3]. Although the majority of the mechanisms related to intracellular Ca^2+^ transport have been successfully elucidated either *in vitro* or in cultured cells, we still know little regarding the actual physiological role of these processes in the context of the tumor environment primarily due to technical limitations. However, recent advancements in imaging have allowed researchers to visualize transient changes in Ca^2+^ levels in live mice [4].

We recently demonstrated that tumor suppressors could modulate cell sensitivity to apoptosis by regulating Ca^2+^ transfer from the endoplasmic reticulum (ER) to mitochondria [5-7]. However, this intriguing signaling pathway has never been investigated in the tumor environment.

This study is the first to demonstrate the direct measurements of intracellular Ca^2+^ dynamics *in vivo* within tumor masses. Importantly, the methodology used in the present study is readily applicable to all the currently available fluorescent probes for following any intracellular parameter of interest. Finally, in the present study, we demonstrate Ca^2+^-dependent p53-mediated apoptosis induced by phototherapy in cancer cells.

This approach will generate new opportunities for elucidating chemoresistance signaling in tumors and for developing new anti-cancer therapy.

## RESULTS

We adopted the well-known tumor suppressor p53 to generate a tumor model related to the loss of an onco-suppressor [8, 9]. The mechanism of action of p53 is dependent on its transcription factor activity. Recently, a cytoplasmic fraction of p53 was suggested to directly modulate apoptosis *via* its cytoplasmic localization [10], which is a common requirement for tumor suppressors that can regulate ER-to-mitochondria Ca^2+^ transfer [6, 7, 11-13].

**Figure 1:**
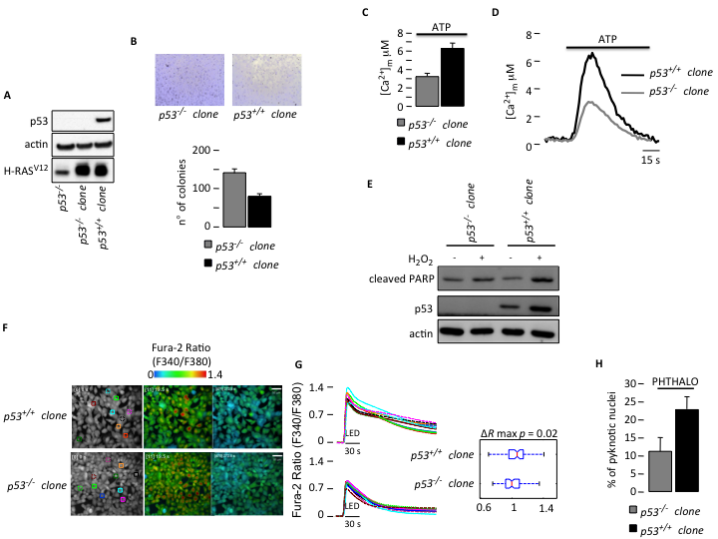
Defective Ca^2+^ homeostasis and apoptosis due to a lack of p53 (A) Generation of H-RAS^v12^-transduced MEF clones. (B) Anchorage-independent growth of H-RAS^v12^-transduced MEFs (*p53^+/+^* clone and *p53^−/−^* clone). Pictures in the upper part are representative of colonies formed. For each well all colonies larger then 0.1 mm in diameter were counted using ImageJ software. (C) Mitochondrial Ca^2+^ response obtained with a mitochondria-targeted aequorin chimera after agonist stimulation with ATP (100 μM) (*p53^+/+^* clone: [Ca^2+^]_m_ peak 6.23 ± 0.44; *p53^−/−^* clone: [Ca^2+^]_m_ peak 2.99 ± 0.21) (p < 0.01). (D) Representative traces of mitochondrial Ca^2+^ transient. (E) Apoptotic sensitivity of H-RAS^v12^-transduced MEF clones (500 μM H_2_O_2_ for 6 hours). (F) Cytosolic Ca^2+^ response of H-RAS^v12^-transduced MEF clones loaded with Fura-2 dye upon phthalocyanine (15 μM) photo-activation with visible (red) light from a 650 nm light emitting diode (LED). Microscopic fields of analyzed cells. (G) The ratio of Fura-2 fluorescence 340 nm/380 nm averaged with the color-matched regions of interest (ROIs) shown in (F), accompanied by statistical analysis. (H) Cell death analysis using the percentage of pyknotic nuclei in H-RAS^v12^-transduced MEF clones upon phthalocyanine (15 μM) photo-activation.

We generated the following two stable clones to verify the relevance of the modulation of Ca^2+^ homeostasis by p53 in the tumor environment: the mouse embryo fibroblast (MEF) *p53^−/−^* clone that was transduced with H-RAS^V12^ (*p53^−/−^* clone) and the MEF *p53^−/−^* clone that was transduced with H-RAS^V12^ with re-introduced wild type (wt) p53 (*p53^+/+^* clone) (Fig. 1A). We tested the anchorage-independent growth, Ca^2+^ response and apoptotic sensitivity of these clones *in vitro*. As expected, the *p53^−/−^* clone formed approximately twice as many colonies in soft agar (Fig. 1B). Moreover, the *p53^−/−^* clone displayed lower mitochondrial Ca^2+^ uptake after agonist stimulation, as analyzed by aequorin technology [14], compared to that of the *p53^+/+^* clone (Fig. 1C and D). This result indicates that p53 modulates intracellular Ca^2+^ homeostasis, presumably by blocking the Ca^2+^ responses generated from the ER, which is a condition that is known to be sufficient to reduce apoptotic sensitivity [15]. Thus, we tested the sensitivity of these two clones to a Ca^2+^-dependent apoptotic stimulus, H_2_O_2_. As consequence of less Ca^2+^ mobilization, the *p53^−/−^* clone exhibited protection from apoptosis when compared to the MEFs clone expressing wt p53 (Fig. 1E). These results suggested that the efficacy of the apoptotic Ca^2+^ signals is p53-dependent.

The next step was to identify a stimulus that could induce an apoptotic Ca^2+^ signal within tumor masses using a drug currently applied in clinical cancer therapy. We selected the anti-cancer photosensitizer aluminum phthalocyanine chloride (phthalocyanine) for this purpose. Phthalocyanine is a light-activatable agent used in cancer photodynamic therapy (PDT) [16]. PDT combines a drug called a photosensitizer or photosensitizing agent with a specific type of light to kill cancer cells preferentially because these cells are more prone to accumulate this drug. Indeed, when the photosensitizer drug in tumors absorbs the light, an active form of oxygen is produced that destroys nearby cancer cells [17]. Several photosensitizers have been shown to localize to intracellular organelles, including the ER, where these photosensitizers promote reactive oxygen species (ROS)-mediated cell death upon illumination with suitable wavelengths of visible (red) light [18].

Because different pro-oxidant agents (*i.e.*, H_2_O_2_ and menadione) can produce Ca^2+^-dependent cell death [5], we hypothesized that phthalocyanine should also produce a Ca^2+^ signal that results in a toxic effect. Thus, we analyzed the sensitivity of these clones to phthalocyanine *in vitro*.

Phthalocyanine photo-activation with a LED light resulted in a rapid increase in the cytosolic Ca^2+^ concentration ([Ca^2+^]_c_) that slowly returned to basal levels, as measured using the ratiometric Ca^2+^ indicator Fura-2. Interestingly, the *p53^−/−^* clone had an impaired Ca^2+^ response (Fig. 1F and G, Videos S1 and S2) and were more resistant in terms of cell death upon phthalocyanine treatment compared to the *p53^+/+^* clone, as indicated by the number of pyknotic nuclei appearing after LED irradiation (Fig. 1H).

**Figure 2:**
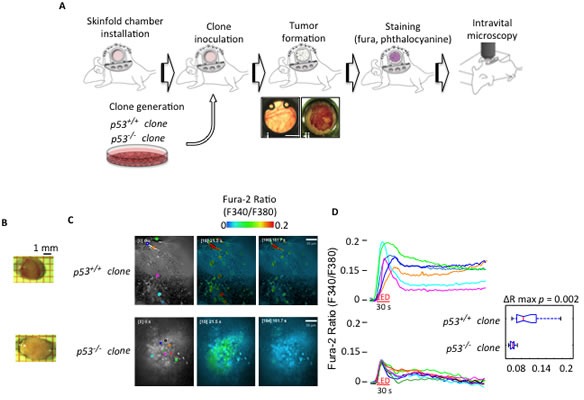
Intravital Ca^2+^ imaging in tumor masses (A) Schematic representation of the skinfold chamber technique used to allow tumor formation and subsequent analysis by ratiometric confocal spinning disk intravital microscopy. (B) Representative images of tumor masses originated by the indicated H-RAS^v12^-transduced MEF clones (*p53^+/+^* and *p53^−/−^* clones) injected in athymic mice. (C) Cytosolic Ca^2+^ response measured within the tumors obtained by H-RAS^v12^-transduced MEF clones injected into skinfold chambers mounted on athymic mice. Microscopic fields of analyzed cells. (D) The ratio of Fura-2 fluorescence 340 nm/380 nm accompanied by statistical analysis.

Next, we tested the involvement of p53 in the generation of pro-apoptotic Ca^2+^ signals evoked by cancer PDT *in vivo*. We developed a new intravital fluorescent microscopy technique to investigate Ca^2+^ signaling in single cells in tumor masses in live mice during PDT. The MEFs of the two clones were injected either subcutaneously or into a skinfold chamber [19] placed on the dorsal skin of athymic mice [20] (Fig. 2A). Subcutaneous tumors were excised after 2 weeks, and as expected, the *p53^−/−^* clone developed tumors that were double in size compared with the tumors that developed from *p53^+/+^* clone (Fig. 2B). Tumors in the dorsal skinfold chamber were loaded with phthalocyanine and the ratiometric Ca^2+^ indicator Fura-2. Ca^2+^ signaling was monitored in live mice using a spinning disk confocal microscope (Fig. 2C). Live ratiometric Ca^2+^ images within the tumor mass and representative traces of the cytosolic Ca^2+^ response after phthalocyanine photo-activation are shown in Fig. 2C and D and in Videos S3 and S4. Higher Ca^2+^ responses were evoked in the tumor mass that developed from the *p53^+/+^* clone compared to those from the tumor mass that developed from the *p53^−/−^* clone (Fig. 2C and D, Videos S3 and S4).

These results strongly suggest that the absence of a functional p53 precludes the generation of an efficient Ca^2+^ response during chemotherapeutic PDT that subsequently prevents the induction of apoptosis to limit tumor growth.

**Figure 3:**
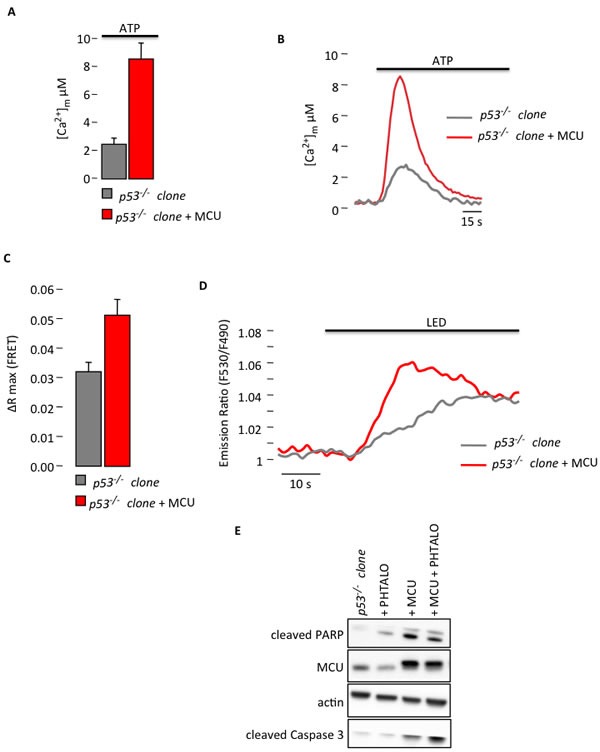
Increased Ca^2+^ response in *p53^−/−^* MEF clones after MCU overexpression restores sensitivity to PDT (A) Mitochondrial Ca^2+^ response after agonist (100 μM ATP) stimulation of *p53^−/−^* MEF clone in resting condition or after MCU overexpression (*p53^−/−^* clone: [Ca^2+^]_m_ peak 2.20 ± 0.45; *p53^−/−^* clone + MCU: [Ca^2+^]_m_ peak 8.28 ± 1.45) (p < 0.01). (B) Representative traces of mitochondrial Ca^2+^ transient. (C) and (D) Single-cell FRET measurements of PDT-induced mitochondrial Ca^2+^ uptake that are expressed as the maximal variation in the emission ratio (*p53^−/−^* clone: ΔR max 0.0311 ± 0.0043; *p53^−/−^* clone + MCU: ΔR max 0.0512 ± 0.0054) (p < 0.05). (E) Immunoblotting for typical apoptotic markers in *p53^−/−^* clone upon phthalocyanine (15 μM) photo-activation (PHTALO).

**Figure 4:**
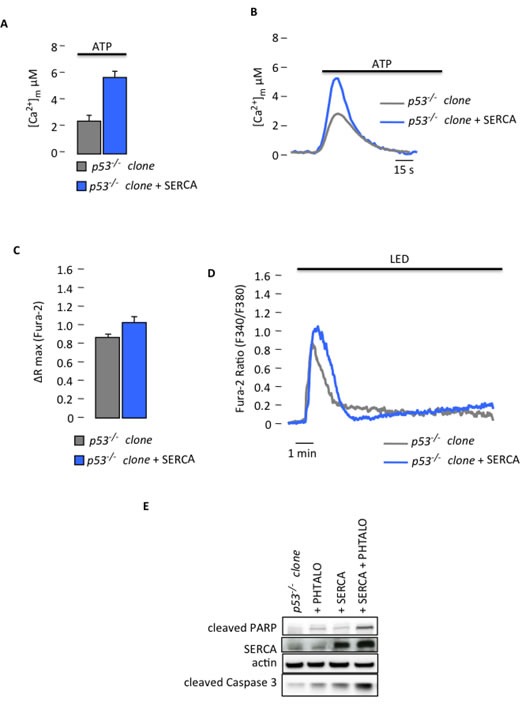
Increased Ca^2+^ response in *p53^−/−^* MEF clones after SERCA overexpression restores sensitivity to PDT (A) Mitochondrial Ca^2+^ response after agonist (100 μM ATP) stimulation of *p53^−/−^* clone in resting condition or after SERCA overexpression (*p53^−/−^* clone: [Ca^2+^]_m_ peak 2.51 ± 0.31; *p53^−/−^* clone + SERCA: [Ca^2+^]_m_ peak 5.40 ± 0.56) (p < 0.01). (B) Representative traces of mitochondrial Ca^2+^ transient. (C) and (D) Ratiometric single-cell measurements of PDT-induced Ca^2+^ waves by Fura-2 that are expressed as the maximal variation in the excitation ratio (*p53^−/−^* clone: ΔR max 0.85 ± 0.02; *p53^−/−^* clone + SERCA: ΔR max 1.05 ± 0.06) (p < 0.01). (E) Immunoblotting for typical apoptotic markers in *p53^−/−^* clone upon phthalocyanine (15 μM) photo-activation (PHTALO).

To demonstrate this hypothesis, we modulated the Ca^2+^ response *in vitro* and *in vivo* to confirm the relation between p53-dependent differences in Ca^2+^ handling and apoptotic behavior. Because we determined that the *p53^−/−^* MEF clones have a reduced response to PDT due to reduced Ca^2+^ signaling, we used two different genetic strategies to restore the Ca^2+^ response and apoptosis: i) overexpression of the mitochondrial Ca^2+^ uniporter (MCU) [21] and ii) overexpression of sarco-ER Ca^2+^-ATPase pumps (SERCA) [22]. The overexpression of the MCU construct in the *p53^−/−^* clone increased the ability of mitochondria to accumulate Ca^2+^. Indeed, the [Ca^2+^]_m_ rise evoked by agonist stimulation and measured using aequorin technology was increased in the *p53^−/−^* clone overexpressing MCU compared to the *p53^−/−^* clone (Fig. 3A and B). These increased values were comparable to those observed in the *p53^+/+^* clone (Fig. 1C), which is consistent with previous publications [23, 24]. We then analyzed the effect of MCU overexpression after PDT. Interestingly, PDT induced a Ca^2+^ response also in the mitochondrial matrix. In fact, cells transfected with the Ca^2+^-sensitive FRET-based probe 2mtD3cpv [25] and treated with phthalocyanine displayed a progressive increase in the FRET ratio (proportional to the mitochondrial Ca^2+^ concentration) after photo-activation-induced Ca^2+^ waves. Furthermore, the stimulated Ca^2+^ uptake by mitochondria was significantly increased after MCU introduction (Fig. 3C and D). This increased mitochondrial Ca^2+^ responsiveness was associated with a re-established apoptotic sensitivity induced by PDT *in vitro* (Fig. 3E). As additional approach we used the overexpression of SERCA. Indeed its increased activity, and the subsequent ER Ca^2+^ overload have been previously demonstrated to promote apoptosis [5, 26]. In our studies, the overexpression of SERCA pumps in the *p53^−/−^* clone restored the mitochondrial Ca^2+^ response after agonist stimulation (Fig. 4A and B) as in the *p53^+/+^* clone (Fig. 1C). Similar to the results obtained after MCU overexpression, also PDT-evoked Ca^2+^ release from the ER into the cytosol was increased (Fig. 4C and D), as measured using the ratiometric Ca^2+^ indicator Fura-2. Consequently, apoptotic sensitivity was rescued in the cells overexpressing SERCA pumps (Fig. 4E). These results support the evidence that the alteration of Ca^2+^ homeostasis observed in the absence of p53, is determinant for sensitivity to apoptosis and that modulating mitochondrial Ca^2+^ handling is possible to re-sensitize cells to death.

**Figure 5:**
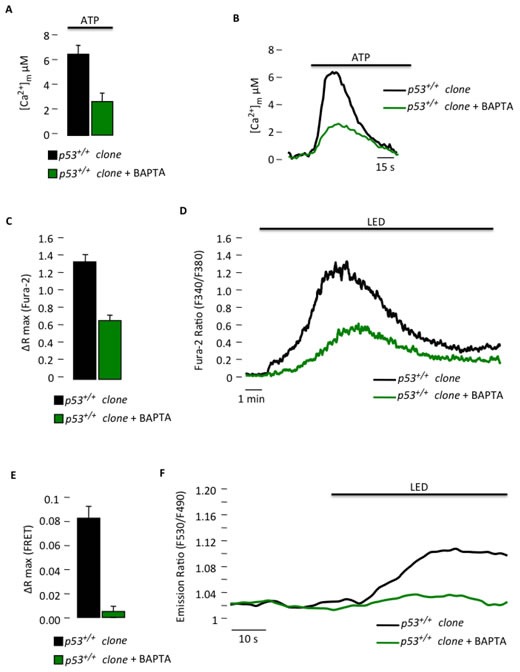

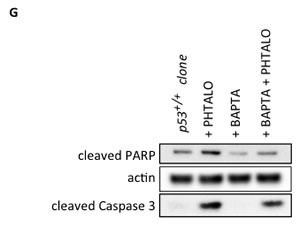
Reduced Ca^2+^ response in *p53^+/+^* MEF clones with a Ca^2+^ chelator blocks the sensitivity to PDT (A) Mitochondrial Ca^2+^ response after agonist (100 μM ATP) stimulation of *p53^+/+^* clone in resting condition or after BAPTA-AM loading (*p53^+/+^* clone: [Ca^2+^]_m_ peak 6.33 ± 0.97; *p53^+/+^* clone + BAPTA: [Ca^2+^]_m_ peak 2.59 ± 0.83) (p < 0.05). (B) Representative traces of mitochondrial Ca^2+^ transient. (C) and (D) Ratiometric single-cell measurements of PDT-induced Ca^2+^ waves by Fura-2 that are expressed as the maximal variation in the excitation ratio (*p53^+/+^* clone: ΔR max 1.35 ± 0.07; *p53^+/+^* clone + BAPTA: ΔR max 0.63 ± 0.04) (p < 0.01). (E) and (F) Single-cell FRET measurements of PDT-induced mitochondrial Ca^2+^ uptake that are expressed as the maximal variation in the emission ratio (*p53^+/+^* clone: ΔR max 0.0824 ± 0.0102; *p53^+/+^* clone + BAPTA: ΔR max 0.0048 ± 0.0045) (p < 0.01). (G) Immunoblotting for typical apoptotic markers in *p53^+/+^* clone upon phthalocyanine (15 μM) photo-activation (PHTALO).

Moreover, we used an approach for preventing Ca^2+^ signaling [27] in the *p53^+/+^* clone to mimic the Ca^2+^ signaling conditions observed both *in vitro* and *in vivo* in the *p53^−/−^* clone to test the dependency of PDT-induced apoptosis on mitochondrial Ca^2+^ uptake. Using BAPTA-AM, which is a well-known membrane permeable Ca^2+^ chelator, we were able to reduce the [Ca^2+^]_m_ response evoked by agonist stimulation (Fig. 5A and B) to values similar to those observed in the *p53^−/−^* clone (Fig. 1C). We further assessed the effect of the chelator after PDT treatment on either the cytosolic (Fig. 5C and D) or mitochondrial Ca^2+^ response (Fig. 5E and F). The reduced ability of mitochondria to accumulate Ca^2+^ was associated with reduced sensitivity to PDT-induced apoptosis (Fig. 5G) as observed previously in the *p53^−/−^* clone (Fig. 1H), probably due to a defective opening of the mitochondrial permeability transition pore [27, 28].

**Figure 6:**
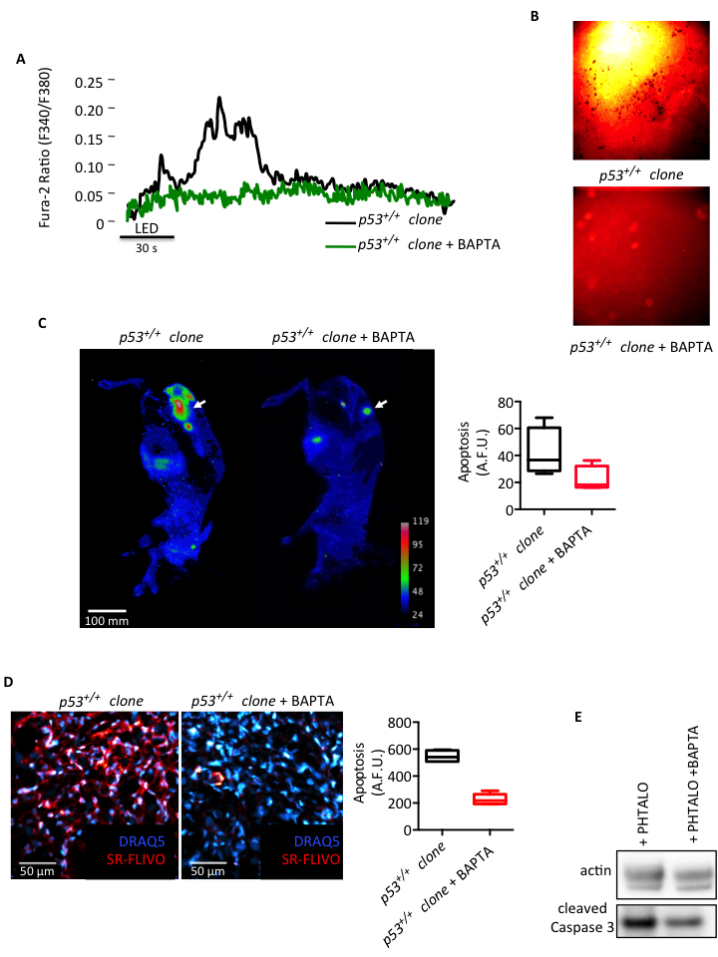
Ca^2+^ signal in tumor masses is required for the anticancer effect of PDT (A) Cytosolic Ca^2+^ response measured within the tumors in the skinfold chambers as the ratio of Fura-2 fluorescence 340 nm/380 nm induced upon phthalocyanine (15 μM) photo-activation. (B) Levels of apoptosis measured as caspase activity (SR-FLIVO) within the tumor masses in the skinfold chambers. (C) *In vivo* imaging of apoptosis as the intensity of fluorescence (SR-FLIVO) emitted by a subcutaneous tumor mass upon phthalocyanine (15 μM) photo-activation. (D) Analysis of apoptosis in tumor tissue sections prepared upon *in vivo* phthalocyanine (15 μM) photo-activation and tumor excision. (E) Immunoblotting of homogenized tumors excised upon *in vivo* phthalocyanine (15 μM) photo-activation.

Then, we also validated our findings *in vivo*. First, we directly verified in the tumor masses (derived from the *p53^+/+^* clone) grown in skinfold chambers and found that the activation of phthalocyanine was unable to induce a significant increase in Ca^2+^ concentration in the presence of BAPTA (Fig. 6A). Then, we directly linked Ca^2+^ response with apoptotic behavior using SR-FLIVO, which is a fluorescent probe used to detect apoptosis *in vivo*. Indeed, as shown in the lower panel of Fig. 6B, tumors treated with PDT exhibited reduced levels of caspase activity in the presence of BAPTA compared to levels of apoptosis in tumors not exposed to this Ca^2+^ chelator (Fig. 6B, upper panel). To further investigate the correlation between Ca^2+^ and apoptosis *in vivo,* two groups of mice were injected subcutaneously with the *p53^+/+^* clones to induce tumor formation. After 14 days, the animals were pre-treated with a control (vehicle) or with BAPTA-AM for 1 hour at the level of the tumor nodules, irradiated with PDT and then injected with SR-FLIVO to image caspase-positive tumor cells. Fig. 6C clearly shows that the inhibition of Ca^2+^ signals reduced apoptotic activation *in vivo* (as previously observed *in vitro*). Finally, tumor nodules were excised and imaged by confocal microscopy (Fig. 6D) or homogenized for Western blot analysis (Fig. 6E). Tumor cells treated with BAPTA displayed significantly decreased fluorescence (and thus, reduced apoptosis due to the inhibition of caspase activation) compared to that of the tumors not treated with BAPTA (*p53^+/+^* clone). These results were confirmed by immunoblotting the extracted tumors with anti-caspase 3 antibody (Fig. 6E).

Taken together, these results indicated that the de-regulation of Ca^2+^ signaling in the absence of p53 reduces the sensitivity of tumor cells to PDT-induced apoptosis.

## DISCUSSION

Our ability to elucidate the details of intracellular signaling has improved remarkably in recent years. Technological innovations resulting from the development of new specialized optical microscopes and from the introduction of fluorescent indicators and proteins allow us to visualize events within a cell in real time and space [14]. These tools provide high sensitivity and great versatility while minimally perturbing the cell under investigation.

Much of the recent progress in understanding the biology of cancer cells has been achieved *via* the study of key components of signaling and regulatory pathways. A widely studied signal transduction pathway in cancer cells is intracellular Ca^2+^ homeostasis [15]. Ca^2+^ plays roles in both tumor progression and the efficacy of chemotherapeutic agents [1], and the participation of this cation in the apoptotic process has been extensively demonstrated [16]. The link of Ca^2+^ to cancer and apoptosis was initially accepted with the discovery that the classical antiapoptotic protein Bcl-2 affected Ca^2+^ signaling. In particular, the antiapoptotic members of the Bcl-2 family cause a reduction in the ER Ca^2+^ concentration, while the opposite is true for proapoptotic proteins. Indeed, apoptotic sensitivity correlates with the total ER Ca^2+^ load and depends on the ability of cells to transfer Ca^2+^ from the ER to the cytosol [17]. Strikingly, proteins other than the proapoptotic members of the Bcl-2 family act by modulating Ca^2+^ homeostasis. Indeed, in recent years, many other onco-suppressor proteins have been shown to reduce the Ca^2+^ release in the ER (albeit through completely different molecular mechanisms) and, consequently, to block the apoptotic responses [5,6,18-20]. However, all of these mechanisms have been successfully elucidated *in vitro*. In contrast, no data regarding the role of intracellular Ca^2+^ homeostasis are available in the context of the tumor environment *in vivo* in mice.

In the present study, we combined the dorsal skinfold chamber technique with intravital microscopy to elucidate the involvement of p53 in the control of intracellular Ca^2+^ signals and apoptosis in three-dimensional tumor masses in living mice. The obtained data strongly indicate that p53-dependent dysregulated Ca^2+^ homeostasis causes reduced ER Ca^2+^ release that is associated with reduced responsivity to apoptotic stimulation. More interestingly, we showed that Ca^2+^ signals with amplitudes that are directly dependent on the presence of p53 and that are tightly linked with the generation of apoptosis in cancer cells are generated when the anti-cancer photosensitizer phthalocyanine, which is used in the photodynamic therapy of cancer, is activated.

To our knowledge, this study is the first to report the direct measurements of intracellular Ca^2+^ dynamics *in vivo* within tumor masses under physiological conditions. Moreover, this imaging approach can be applied successfully to all the various GFP moieties and chimeras, as well as to all of the fluorescent probes that are available, to follow intracellular parameters of interest.

Overall, these results provide new insights into p53 pathways underlying cancer and a method to monitor the kinetics of intracellular signals in tumor masses *in vivo* to verify the efficacy of anticancer treatments.

## MATERIALS AND METHODS

### Reagents and solutions

ATP, histamine, digitonin, H_2_O_2_, and phthalocyanine were purchased from Sigma-Aldrich. Coelenterazine was purchased from Synchem. Fura-2 AM and BAPTA-AM were purchased from Invitrogen.

Krebs-Ringer bicarbonate solution (KRB) contained 125 mM NaCl, 5 mM KCl, 1 mM MgSO_4_, 1 mM Na_2_HPO_4_, 5.5 mM glucose, 20 mM NaHCO_3_, 2 mM l-glutamine and 20 mM HEPES (pH 7.4) and was supplemented with 1 mM CaCl_2_.

### Cell culture, transfection and cell death induction

HeLa cells were obtained from the American Type Culture Collection. The HeLa cells were cultured in Dulbecco's modified Eagle's medium (DMEM) supplemented with 10% FCS and transfected using a standard calcium phosphate procedure.

The MEFs were generated by crossing *p53^+/−^* mice and collecting cells from 13.5 d.p.c. embryos. The MEFs were cultured in DMEM supplemented with 10% FCS and treated with 500 μM H_2_O_2_ for 6 hours or with 15 μM phthalocyanine for 2 hours in complete medium.

### Detection of cell death

*In vitro*. Apoptosis was determined by different methods as indicated in the text: (i) by blotting for cell death markers, such as cleaved PARP or cleaved Caspase 3, or (ii) by the automated detection of pyknotic nuclei. Briefly, cells were fixed and stained with DAPI, and images were acquired using an automated microscope as previously described. Then, pyknotic nuclei were identified by combining DAPI intensity and the nucleus form factor.

*In vivo*. After an i.v. injection of 100 μl of SR-FLIVO™ (Immunochemistry Technologies) *via* the lateral tail vein, the FLIVO reagent was allowed to circulate in the mouse for 30 minutes before analysis. Fluorescent *in vivo* images were acquired using an Odessey® CLx Imaging System (Li-Cor). Then, the tumors were excised, frozen, sectioned and stained for nuclei using DRAQ5 according to the manufacturer's protocol (Cell Signaling). After staining, the samples were mounted on coverslips and analyzed using a Zeiss LSM 510 confocal microscope equipped with a Fluor 40x/1.30 oil immersion objective. The acquired images were background corrected, and signals were analyzed using the open source Fiji software (available at http://fiji.sc/Fiji).

### Aequorin measurements

Cells grown on 13 mm round glass coverslips at 50% confluence were transfected with the appropriate chimera cytAEQ or mtAEQ as previously described [7] alone or together with p53 wt expression constructs.

All aequorin measurements were performed in KRB. Agonists were added to the same medium as specified in the figure legends. The experiments were terminated by lysing the cells with 100 μM digitonin in a hypotonic Ca^2+^-rich solution (10 mM CaCl_2_ in H_2_O), thus discharging the remaining aequorin pool. The light signals were collected and calibrated to [Ca^2+^] values as previously described [7].

### Fura-2 measurements

The cytosolic Ca^2+^ response was evaluated using the fluorescent Ca^2+^ indicator Fura-2/AM (Life Technologies, Invitrogen). The MEFs were grown on 24-mm coverslips and incubated at 37 °C for 30 minutes in 1 mM Ca^2+^/KRB supplemented with 2.5 mM Fura-2/AM, 0.02% Pluronic F-68 (Sigma-Aldrich), and 0.1 mM sulfinpyrazone (Sigma-Aldrich). Then, the cells were washed and supplied with 1 mM Ca^2+^/KRB. Next, the cells were placed in an open Leyden chamber on a 37 °C thermostated stage and exposed to 340/380 wavelength light using the Olympus xcellence (Olympus) multiple wavelength high-resolution fluorescence microscopy system equipped with an Hamamatsu ORCA ER CCD camera (Hamamatsu Photonics) and a Upl FLN 40x oil objective (Olympus) to determine the cytosolic Ca^2+^ response. The photo-activation of aluminum phthalocyanine chloride was obtained using an excitation filter ET576/25 (Semrock), with 500 milliseconds of excitation every FRET ratio cycle. The collected fluorescence data are expressed as emission ratios.

### FRET-based mitochondrial Ca^2+^ measurements

MEF cells were grown on 24-mm coverslips transfected with 4mtD3cpv. After 36 hours, the cells were imaged using a Zeiss Axiovert 200M microscope with a cooled CCD camera (Photometrics), which was equipped with a C-apochromatic 40x/1.2 W CORR objective and controlled by MetaFluor 7.0 software (Universal Imaging). Emission ratio imaging of 4mtD3cpv was accomplished using a 436DF20 excitation filter, a 450 nm dichroic mirror, and two emission filters (475/40 for ECFP and 535/25 for citrine) that were controlled by a Lambda 10-2 filter changer (Sutter Instruments, Novato, CA 94949, USA). The acquired fluorescence images were background corrected. The exposure times were typically 100–200 milliseconds, and images were collected every second per wavelength. The photo-activation of aluminum phthalocyanine chloride was achieved using an excitation filter ET650/50 (Chroma Technology Corp.), with 500 milliseconds of excitation every FRET ratio cycle.

### Transformation assays and *in vivo* tumorigenicity

Low-passage MEF clones infected with H-RAS^V12^ or H-Ras^V12^ + p53 wt were resuspended in DMEM that was supplemented with 10% fetal bovine serum, penicillin (100 IU/ml) and streptomycin (100 IU/ml) and that contained 0.3% agarose. Then, the cells were plated on top of 1% agarose in the same medium at a cell density of 1 × 10^5^ for each 60 mm plate. Colonies reaching at least 100 μm were scored after 14 days in culture. These experiments were performed in triplicate.

To assess the tumorigenicity of H-RAS^V12^-infected MEFs, 1 × 10^6^ cells were injected subcutaneously on both sides of athymic mice, and tumors were excised after 2 weeks. Procedures involving animals and their care conformed to institutional guidelines, and all experimental protocols were approved by the Animal Ethics Committee.

### Dorsal skinfold chamber and intravital microscopy

Dorsal skin-fold chambers were transplanted onto 7-week-old female athymic mice (Harlan), as described [8]. A cell pellet containing 10^6^ tumor cells was placed onto the tissue surface (drop-on method) at three days post-surgery. Intravital microscopy analyses were performed two weeks after cell inoculation as follows: the mice were anesthetized by halothane and transferred to a custom-made warmed plate mounted on the stage of an upright spinning disk confocal system microscope (Olympus DSU) equipped with a water immersion objective (Fluor 40x, 1.0 N.A., Nikon) for observation. The AM ester of Fura-2 (15 μM) and aluminum phthalocyanine chloride (dissolved in DMS at a final concentration of 15 μM) were loaded into each tumor mass by pressure injection with a glass microcapillary formed on a pipette puller (PP-830, Narishige). Cytosolic Ca^2+^ signals were tracked in the dorsal skinfold chamber by the sequential illumination of Fura-2 at 340 nm and 380 nm from two light emitting diodes. Fluorescence images were formed on the scientific complementary metal–oxide–semiconductor (sCMOS) sensor of a high-performance camera (pco.edge) under the control of software written in the laboratory. Images were acquired *in vivo* before, during and after the photo-activation of aluminum phthalocyanine chloride with light from a 650 nm light emitting diode placed immediately above the microscope objective and directed onto the sample by reflection off a dichromatic short-pass mirror (Edmund Optics, part n. 69-217). The acquired images were processed off-line by software written in the laboratory using the Matlab environment (Release 14, The MathWorks, Inc., Natick, MA, USA). Signals were measured as dye emission ratio changes using the following equation: Δ*R* = *R*(t) − *R*(0), where *t* is time, *R*(*t*) is the emission intensity excited at 340 nm divided by the intensity excited at 380 nm, and *R*(0) indicates the pre−stimulus ratio.

For the BAPTA-AM experiments, 1 μM BAPTA was loaded together with Fura-2 and phthalocyanine into each tumor mass. For the *in vivo* detection of apoptosis in the dorsal skinfold chamber, SR-FLIVO (see previous paragraph) was injected *in situ* and incubated for 60 minutes in the exposed area, and excess fluorophore was extensively washed with PBS. Then, images were acquired using the previously described imaging system, which was equipped with a Zeiss EC Plan-Neo fluor 5x/0.16 objective.

### Statistical analysis of data

The statistical data are presented as the mean ± SE. Significance was calculated by Student's t-test, and correlation analysis was conducted using SigmaPlot 5.0 software (SPSS Inc.).

## SUPPLEMENTARY MATERIALS AND VIDEOS










