# Supplementary material for: Intravital imaging reveals p53-dependent cancer cell death induced by phototherapy via calcium signaling
Source: Oncotarget. 2014 Dec 2;6(3):1435–45. doi: 10.18632/oncotarget.2935 (PMC4359305; doi:10.18632/oncotarget.2935)
Supplement: Supplementary file 5 [file oncotarget-06-1435-s005.pdf]

Intravital imaging reveals p53-dependent cancer cell death induced by phototherapy via calcium signaling

Supplementary Material

Video S1: *In vitro* Ca^{2+} waves registered in the *p53*$^{+/+}$ clone upon phthalocyanine photo-activation.

Video S2: *In vitro* Ca^{2+} waves registered in the *p53*$^{-/-}$ clone upon phthalocyanine photo-activation.

Video S3: *In vivo* Ca^{2+} waves registered in a tumor mass obtained with the *p53*$^{+/+}$ clone upon phthalocyanine photo-activation.

Video S4: *In vivo* Ca^{2+} waves registered in a tumor mass obtained with the *p53*$^{-/-}$ clone upon phthalocyanine photo-activation.
